# Acrolein-inducing ferroptosis contributes to impaired peripheral neurogenesis in zebrafish

**DOI:** 10.3389/fnins.2022.1044213

**Published:** 2023-01-12

**Authors:** Haozhe Qi, Kejia Kan, Carsten Sticht, Katrin Bennewitz, Shu Li, Xin Qian, Gernot Poschet, Jens Kroll

**Affiliations:** ^1^Department of Vascular Biology and Tumor Angiogenesis, European Center for Angioscience, Medical Faculty Mannheim, Heidelberg University, Mannheim, Germany; ^2^Department of Vascular Surgery, Renji Hospital, School of Medicine, Shanghai Jiao Tong University, Shanghai, China; ^3^Department of Surgery, Universitätsmedizin Mannheim, Medical Faculty Mannheim, Heidelberg University, Mannheim, Germany; ^4^The Next-Generation Sequencing (NGS) Core Facility, Medical Faculty Mannheim, Heidelberg University, Mannheim, Germany; ^5^Metabolomics Core Technology Platform, Centre for Organismal Studies, Heidelberg University, Heidelberg, Germany

**Keywords:** ferroptosis, diabetic peripheral neuropathy, neurogenesis, neurodegeneration, acrolein

## Abstract

**Introduction:**

Diabetes mellitus (DM) is associated with physiological disorders such as delayed wound healing, diabetic retinopathy, diabetic nephropathy, and diabetic peripheral neuropathy (DPN). Over 50% of diabetic patients will develop DPN, characterized by motor dysfunction and impaired sensory nerve function. In a previous study, we have uncovered acrolein (ACR) as an upstream initiator which induced impaired glucose homeostasis and microvascular alterations in zebrafish. Whether ACR has specific effects on peripheral neurogenesis and mediates DPN, is still waiting for clarification.

**Methods:**

To evaluate the function of ACR in peripheral nerve development, *in vivo* experiments were performed in *Tg(hb9:GFP)* zebrafish. In addition, a series of rescue experiments, metabolomics assessment, and bioinformatics analysis was performed aimed at identifying the molecular mechanisms behind ACR’s function and impaired neurogenesis.

**Results:**

Impaired motor neuron development was confirmed in wild-type embryos treated with external ACR. ACR treated embryos displayed ferroptosis and reduction of several amino acids and increased glutathione (GSH). Furthermore, ferroptosis inducer caused similarly suppressed neurogenesis in zebrafish embryos, while anti-ACR treatment or ferroptosis inhibitor could successfully reverse the detrimental phenotypes of ACR on neurogenesis in zebrafish.

**Discussion:**

Our data indicate that ACR could directly activate ferroptosis and impairs peripheral neurogenesis. The data strongly suggest ACR and activated ferroptosis as inducers and promising therapeutic targets for future DPN studies.

## 1. Introduction

Diabetic peripheral neuropathy (DPN) is recognized as the most common diabetic complication, with more than 50% of diabetic patients who will eventually develop DPN (Pop-Busui et al., 2013; Zakin et al., 2019). Despite such high prevalence, the basic mechanisms of DPN remain partially unknown. DPN manifests mainly as sensory disorders with numbness, allodynia, and pain at an early stage (Pop-Busui et al., 2017). Without effective intervention on time, these primary manifestations will gradually progress to muscle weakness, spastic paralysis, reduced mobility, and increase the risk of disability, ulceration, and amputation caused by degeneration of motor neurons and death of neural stem cells (Feldman et al., 2017; Pop-Busui et al., 2017; Holmes and Hastings, 2021). Hence, early diagnosis and treatment of DPN are urgently needed to improve the life quality of diabetic patients. Clinical treatment for DPN nowadays is still limited to strict hypoglycemic therapy and pain management, while a restricted number of patients benefit from it, which implies more culprits besides hyperglycemia may take part in the processing of DPN (Callaghan et al., 2012; Azmi et al., 2019; Stino et al., 2020).

Increasing evidence indicates that reactive carbonyl species (RCS) are positively correlated with diabetic complications, including but not limited to diabetic retinopathy, nephropathy, and neuropathy (Uchida, 2000; Brownlee, 2001). ACR as one of those factors has drawn extreme attention due to its toxic effects and capacity to react with a wide range of proteins, nucleic acids, and other biological molecules (Lovell et al., 2001; Stevens and Maier, 2008; Murata et al., 2020). Preliminary studies have identified an increase of the ACR-lysine adduct (FDP-lysine) in type 1 and type 2 diabetic patients (Daimon et al., 2003; Tsukahara et al., 2003). FDP-lysine level was confirmed to positively correlate with diabetic nephropathy and retinopathy (Noiri et al., 2002; Zhang et al., 2008). Moreover, in our previous study, elevated ACR in *akr1a1a* zebrafish mutants displayed a harmful effect on glucose homeostasis *via* impairing insulin signaling transduction and induced morphological alterations in retina vessels and the biological structure of kidneys (Qi et al., 2021). Transcriptome data from our previous work also strongly suggested potential connections between ACR and neurogenesis. Therefore, we aimed to address whether ACR is involved in the neurogenesis and the degeneration of motor neurons (Qi et al., 2021).

As a well-established animal model, the zebrafish has widely been used in the study of metabolic diseases and diabetic complications due to its unique advantages in high fecundity, short generation time, and rapid external development of transparent embryos (Heckler and Kroll, 2017; Wiggenhauser and Kroll, 2019). Furthermore, zebrafish are also well-studied for peripheral neuropathy due to the visibility of peripheral motor neurons by using special genetic lines (Gong et al., 2020; Lu et al., 2021). Ennerfelt et al. proved that hyperglycemia disrupted peripheral nerve development in a zebrafish model, suggesting zebrafish as an emerging model for studying DPN (Ennerfelt et al., 2019).

This study aimed to address whether ACR has detrimental effects on the peripheral motor neurons and identify the underlying potential mechanisms. In addition, we tested if anti-ACR treatment would be a promising approach for DPN therapy. Our data indicate that ACR could directly activate ferroptosis and impairs peripheral neurogenesis. Subsequently, anti-ACR and anti-ferroptosis treatment could efficiently reverse the adverse effects of ACR and promote peripheral neurogenesis.

## 2. Materials and methods

### 2.1. Zebrafish husbandry

Zebrafish lines, *Tg(fli1:EGFP)* and *Tg(hb9:GFP)* were raised as previously described under standard husbandry conditions (Kimmel et al., 1995; Lawson and Weinstein, 2002; Nakano et al., 2005). Embryos/larvae were kept in E3 media at 28.5°C with/without PTU (2.5 ml in 25 ml) to suppress pigmentation formation. All experimental procedures performed on zebrafish were approved by the local government and Medical Faculty Mannheim of Heidelberg University.

### 2.2. RNA-seq analysis

Total RNA was isolated from WT and WT & ACR larvae at 120 hpf. Library construction and sequencing were performed with BGISEQ-500 (Beijing Genomic Institution,^1^ BGI). Gene expression analysis were conducted by the Core-Lab for microarray analysis, center for medical research (ZMF). Quality control and data analysis were performed as described previously (Qi et al., 2021). The RNA-Seq datasets produced in this study are available at GEO (Gene Expression Omnibus, NIH) under the accession address: https://www.ncbi.nlm.nih.gov/geo/query/acc.cgi?acc=GSE168786.

### 2.3. Microscopy and analysis of neurogenesis in embryos

For imaging of the zebrafish neurogenesis, *Tg(hb9: GFP)* larvae were anesthetized in 0.0003% tricaine and dechorinated at 24 hpf, 48 hpf, respectively and fixed in 1% low melting gel for scanning. Confocal images for phenotype evaluation were acquired using a confocal microscope (DM6000 B) with a scanner (Leica TCS SP5 DS) utilizing a 20 × 0.7 objective, 1,024 × 1,024 pixels, 0.5 μm Z-steps. The motor axon length at a defined location in the intersomitic segments was measured for 4–6 axons per larva by blinded observer using ImageJ software with neural tracking plugin (Vaccaro et al., 2012; Ciura et al., 2013; Pagnamenta et al., 2021). The axon length was measured in detail (Supplementary Figure 1).

### 2.4. Pharmacological treatment of zebrafish embryos

Fertilized zebrafish embryos were transferred into 6-well plate, about 30 embryos per well with 5 ml eggwater. At 24 hpf the chorion of zebrafish embryos was removed using sharp tweezers and 0.003% PTU was added to the eggwater. For ACR intervention and rescue experiments, 10 μM ACR (S-11030F1; CHEM SERVICE), 10 μM PK11195 (C0424; Sigma-Aldrich), 10 mM L-Carnosine (C9625; Sigma-Aldrich), 10 μM Erastin (E7781; Sigma-Aldrich), and 1 μM Ferrostatin (SML0583; Sigma-Aldrich) treatments were started from 1 hpf and continued until 2 dpf. Medium was refreshed daily. For Ferrostatin treatment a titration curve indicated concentrations above 1 μM as toxic and were therefore excluded from the study (Supplementary Figure 2).

### 2.5. Metabolomic analysis

The measurements were done in cooperation with the Metabolomics Core Technology Platform from the Centre of Organismal Studies Heidelberg. 50 zebrafish larvae at 120 hpf per measurement were snap-frozen in liquid nitrogen. As previously described, adenosine compounds, thiols, and free amino acids were measured (Qi et al., 2021).

### 2.6. Software

For schematic diagrams generation, Biorender^2^ was used. Analysis of neurogenesis was carried out by using LAS AF Lite Software from Leica for taking screenshots, Gimp for image cutting and ImageJ for quantification. The “GCMS solution” software (Shimadzu^®^) was used for data processing of the GC/MS analysis.

### 2.7. Statistical analysis

Sample size for all experiments is more than three independent biological replicates. Data were displayed as mean with standard deviation. Statistical significance between different groups was analyzed using two-paired Student’s *t*-test, one-way ANOVA (followed by Tukey’s multiple comparisons) by GraphPad Prism 6.01 or 8.3.0. Principal Component Analysis was performed by R programming language. *P*-values of 0.05 were considered as significant: *p < 0.05, **p < 0.01, ***p < 0.001, ****p < 0.0001.

## 3. Results

### 3.1. RNA-Seq analysis displayed altered biological patterns in ACR treated zebrafish embryos

**FIGURE 1:**
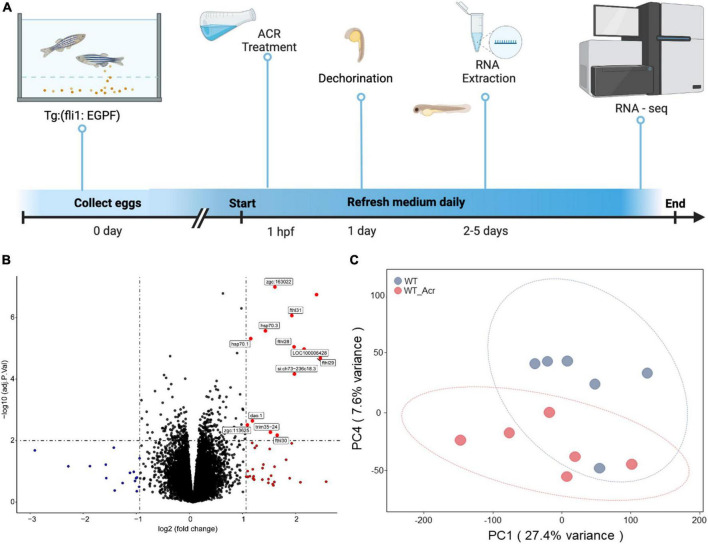
RNA-Seq analysis of wildtype zebrafish with or without acrolein (ACR) treatment at larval stage. **(A)** Scheme of experimental design for larval RNA-Seq. 30 larvae per clutch, 6 clutches of WT and WT & ACR zebrafish larvae at 5 dpf were applied for RNA-Seq analysis. **(B)** Volcano plot showed significantly up-regulated (red dots) genes (logFC > 1, adj.*P*.val < 0.01) in WT & ACR zebrafish larvae. **(C)** Gene expression patterns between WT and WT & ACR zebrafish larvae at 5 dpf were analyzed using PCA. The PC1 and PC4 are represented on the X-axis and Y-axis, respectively. PCA, principal component analysis; PC1, principal component 1; PC4, principal component 4; dpf, day post fertilization.

According to the previous studies, ACR does have a solid connection to neuropathic pain, spinal cord injury, cardiovascular disease, and diabetes (DeJarnett et al., 2014; Butler et al., 2017; Jiang et al., 2018; Lin et al., 2018), but whether ACR has an influence on the neurogenesis in zebrafish and the potential molecular mechanism behind is still far away from clarity. To address this question, we treated wild-type zebrafish embryos with external ACR (Qi et al., 2021), refreshed the medium daily, collected the larvae at 5 dpf, and extracted total RNA for quality test and further RNA-seq analysis (Figure 1A).

**FIGURE 2:**
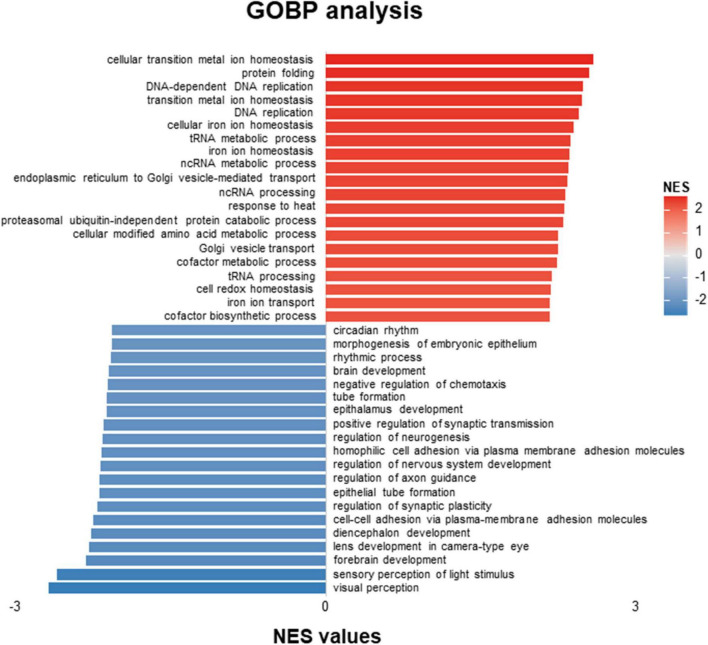
GOBP-analysis of RNA-Seq data showed patterns distinguishing acrolein (ACR) treated zebrafish from controls. Top twenty up-regulated and down-regulated pathways based on GOBP enrichment analysis of RNA-Seq results in WT and WT & ACR zebrafish larvae at 5 dpf. GOBP, Gene Ontology Biological Process; dpf, day post fertilization.

The volcano figure showed that about thirteen genes are significantly up-regulated in the embryos treated with ACR at 5 dpf (Figure 1B). Principal component analysis (PCA) exhibited components of each sample, which showed that wild-type (WT) and wild-type treated with ACR (WT & ACR) plots are most separated in the PC4 axis (Figure 1C). Moreover, gene set enrichment analysis (GSEA) was performed to give a better understanding of altered physiological processes reflected by the treatment with ACR. The twenty most up-regulated and down-regulated biological patterns were filtered and sorted on the basis of the enrichment score (NES, normalized enrichment score). Intriguingly, several iron ion homeostasis pathways, including cellular transition metal ion homeostasis, transition metal homeostasis, cellular iron ion homeostasis, iron ion homeostasis, and iron ion transport, showed positive alteration. In contrast, several neurogenesis-relevant pathways were negatively regulated, such as regulation of neurogenesis, regulation of nervous system development, and regulation of axon guidance (Figure 2). The above data suggest that ACR may restrain neurogenesis, facilitate the iron transition and break iron ion homeostasis.

### 3.2. ACR suppressed motor neuron development in zebrafish embryos

**FIGURE 3:**
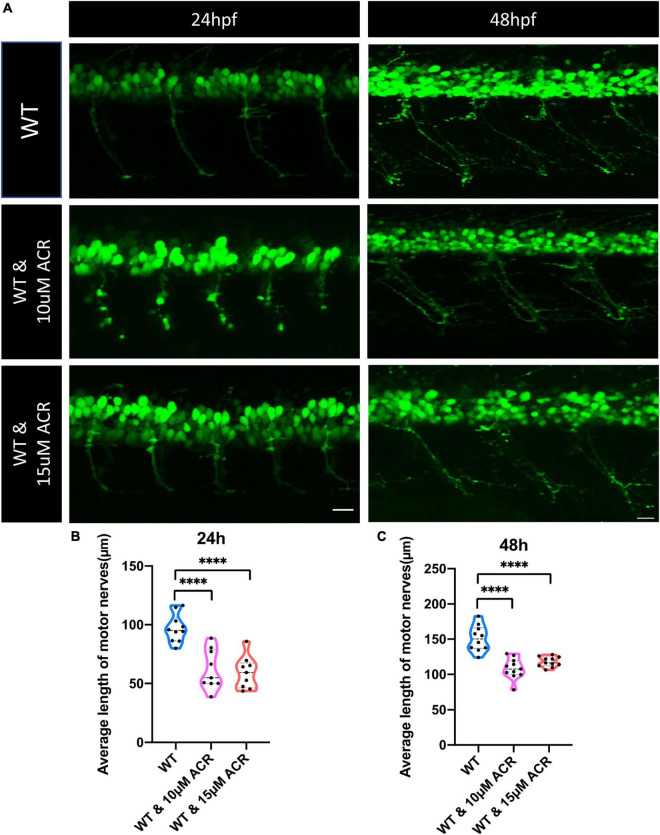
Disrupted motor neuron development in acrolein (ACR) treated zebrafish embryos. **(A)** Representative confocal images of motor nerves. White scale bar: 20 μm. **(B,C)** Quantification of average length of motor nerves showed significant decrease of axonal length in embryos upon ACR treatment at 24 and 48 hpf. *n* = 9–11. For statistical analysis Student’s *t*-test was applied; ****p < 0.0001. hpf, hour post fertilization.

To further confirm the hypothesis that ACR may suppress neurogenesis, *Tg(hb9:GFP)* zebrafish were used for embryo breeding since green fluorescence protein primarily express in motoneurons and a small population of spinal interneurons in this particular line and make it possible to observe the neurogenesis process *in vivo*. The wild-type *Tg(hb9:GFP)* line embryos were incubated with ACR since 1 hpf, and the morphology of motor neuron development was assessed at 24 and 48 hpf, respectively. It was observed that the average length of motor nerves was significantly shorter in WT group treated with ACR as compared to WT group without ACR treatment, which preliminarily proves that ACR suppressed neurogenesis in zebrafish embryos (Figure 3).

**FIGURE 4:**
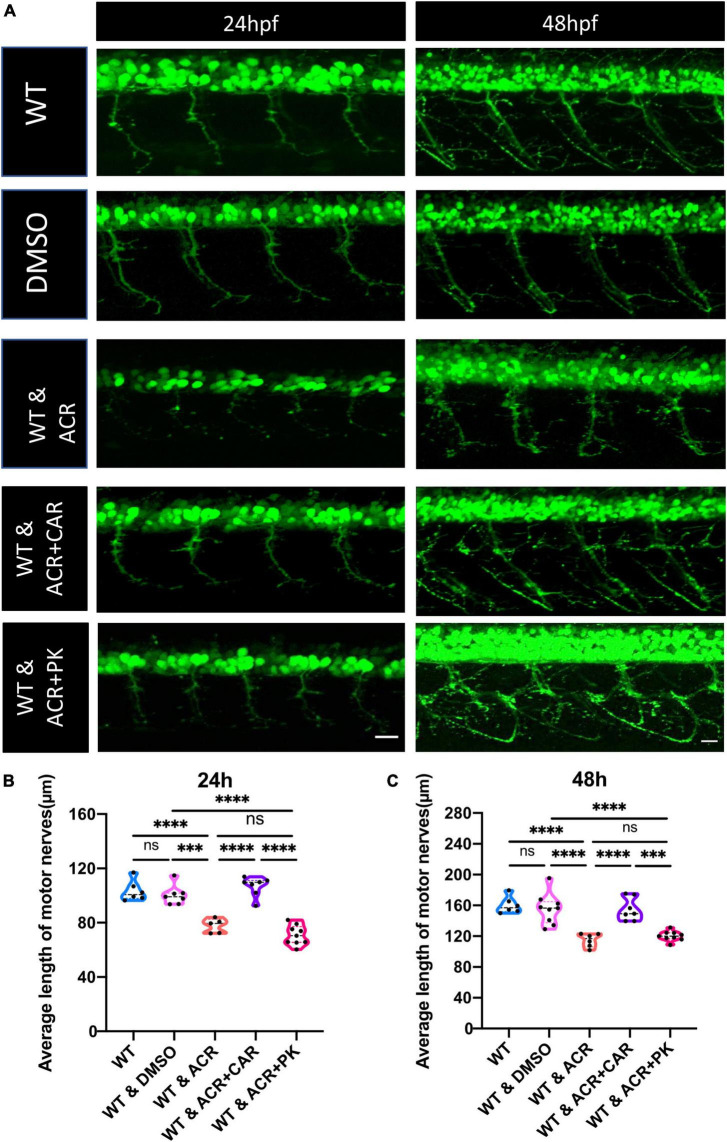
Beneficial effects of anti-acrolein (ACR), but not hypoglycemic treatment on motor neuron development in ACR treated embryos. **(A)** Representative confocal images of motor neurons at 24 and 48 hpf upon different treatment. White scale bar: 20 μm. **(B,C)** Quantification of average length of motor nerves showed significant decrease of axonal length in embryos upon ACR treatment can be well-reversed by L-carnosine (anti-ACR treatment) but not PK11195 (hypoglycemic treatment) at 24 and 48 hpf. *n* = 5–9. For statistical analysis one-way ANOVA followed by Tukey’s multiple comparisons test was applied. ***p < 0.001, ****p < 0.0001. ns, not significant; hpf, hour post fertilization.

According to our previous study, ACR could also destroy the insulin receptor signaling pathway, resulting in impaired glucose homeostasis (Qi et al., 2021). In order to distinguish destructive neurogenesis in ACR treated embryos resulting from impaired glucose homeostasis or ACR itself, anti-ACR drug (L-carnosine) (Zhao et al., 2019; Spaas et al., 2021) and hypoglycemic drug (PK11195) (Wiggenhauser et al., 2020) were utilized. Interestingly, anti-ACR, but not hypoglycemic treatment, could rescue the shortened motor nerves (Figure 4). Thus, ACR could induce impaired neurogenesis directly, and anti-ACR treatment would be a promising approach to rectify motor neuron development.

### 3.3. Imbalanced ferroptosis leads to restrained neurogenesis after ACR treatment

**FIGURE 5:**
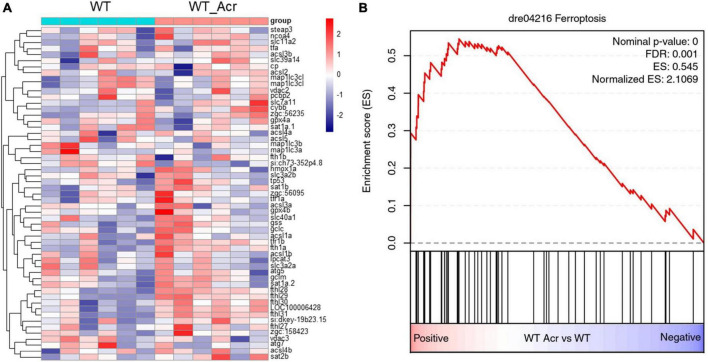
RNA-Seq data displayed up-regulated ferroptosis in acrolein (ACR) treated zebrafish larvae at 5 dpf. **(A)** Heatmap showed relative mRNA expression in ferroptosis biological process. **(B)** RNA-Seq GSEA analysis showed up-regulated expression of ferroptosis in ACR treated zebrafish larvae. The higher and lower expression level is showed in red and purple, respectively. GSEA, gene set enrichment analysis; dpf, day post fertilization.

**FIGURE 6:**
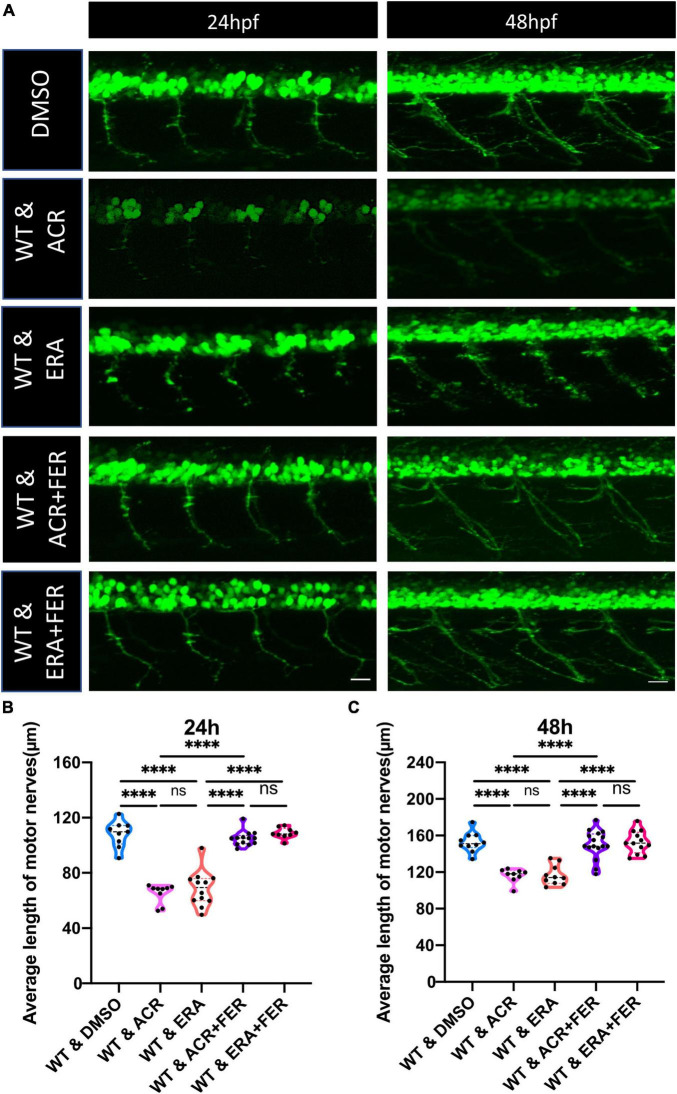
Motor neuron development under ferroptosis inducer and ferroptosis inhibitor treatment. **(A)** Representative confocal images of motor nerves at 24 and 48 hpf upon different drugs treatment. White scale bar: 20 μm. **(B,C)** Quantification of average length of motor nerves showed significant decline of axonal length in embryos upon ferroptosis inducer Erastin treatment. The ferroptosis inhibitor Ferrostatin can reverse the impaired motor neuron development in Erastin and acrolein (ACR) treated embryos at 24 and 48 hpf. *n* = 9–16. For statistical analysis one-way ANOVA followed by Tukey’s multiple comparisons test was applied. ****p < 0.0001. ns, not significant; hpf, hour post fertilization.

Since several iron ion homeostasis pathways showed significant alterations, we considered whether the ferroptosis process may induce notable change. GSEA analysis showed that the ferroptosis process displayed a significant up-regulation, accompanied by relevant genes highly expressed, such as fthl28, fthl29, fthl30, fthl31, and LOC100006428 (Figure 5). To date, numerous studies have proved that ferroptosis plays specific roles in neurogenesis (Cozzi et al., 2019; Song et al., 2022). Therefore, we wondered if imbalanced ferroptosis is the potential mechanism between ACR and impaired neurogenesis. Ferroptosis activator (Erastin) and ferroptosis inhibitor (Ferrostatin) were utilized for further incubation experiments to verify this hypothesis. It proved that Erastin could induce a similar phenotype that was observed in ACR treated zebrafish before, suggesting activated ferroptosis could cause impaired neurogenesis. Furthermore, co-incubated with Ferrostatin, the ACR and Erastin treated embryos showed a regular average length of motor nerves similar to the control group (Figure 6). In case the Ferrostatin may bind and subsequently destruct ACR’s function outside the embryos, we incubated different concentrations of Ferrostatin together with the 10 μM ACR overnight at 28°C and then added the mixtures to the zebrafish embryos. As can be seen in Supplementary Figure 3, there is no significantly altered neurogenesis in this experimental setting. Specifically, the simple administration of ACR pre-incubated overnight at 28°C) did not induce a similar phenotype as observed before (Figures 3–6). Thus, the preincubation process is accompanied by the loss of the biological activity of ACR. In conclusion, the above data support that excessive ferroptosis contributes to the impaired neurogenesis in ACR treated zebrafish embryos.

### 3.4. ACR treatment resulted in metabolic alterations in zebrafish embryos

**FIGURE 7:**
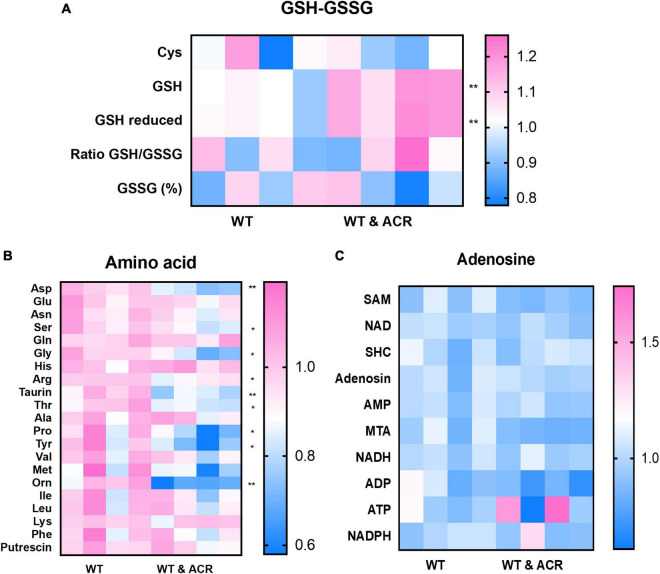
Metabolomic screening displayed several alterations in acrolein (ACR) treated zebrafish larvae at 5 dpf. **(A–C)** Heatmaps showed the metabolomic screening of thiols panel **(A)**, amino acids panel **(B)** and adenosines panel **(C)** in WT and WT & ACR zebrafish larvae at 5 dpf: panel **(A)** Glutathione (GSH) and reduced GSH showed significant increase in WT & ACR zebrafish; panel **(B)**. Several amino acids displayed significant reduction after treatment with ACR; panel **(C)**. No changes in adenosine levels were identified in WT & ACR larvae as compared to WT larvae; *n* = 4 clutches with 50 larvae, for statistical analysis Student’s *t*-test was applied; *p < 0.05, **p < 0.01. dpf, day post fertilization.

To determine the metabolic patterns in ACR treated embryos, we performed targeted metabolomics comparing ACR treated and control embryos. Several thiols, adenosine contents, and amino acids were detected. The results displayed that GSH and reduced GSH increased significantly. Moreover, a series of amino acids showed a dramatic decline in ACR treated embryos, while adenosines did not exhibit any apparent alteration, which implies the activated ferroptosis in ACR treated larvae gives rise to alterations in amino acids and affects GSH metabolism (Figure 7).

## 4. Discussion

Up to now, several processes such as oxidative stress, inflammation, increased activity of polyol pathway, an increase of protein glycosylation products, and disturbance of lipid metabolism are widely recognized as contributing to the development of DPN (Callaghan et al., 2015, 2016, 2018; Sanaye and Kavishwar, 2023). However, the molecules which mediate the onset and progression of neuropathy are still controversial. In a previous study, we have identified that ACR, a side-product of lipid peroxidation, causes diabetic retinopathy and nephropathy *via* destructing glucose homeostasis and inducing endogenous hyperglycemia (Qi et al., 2021), while whether ACR also plays specific roles in DPN and the potential mechanisms involved is still unknown.

In this study, we explored the effects of ACR on peripheral neuropathy in zebrafish for the first time. Based on RNA-Seq data and subsequent *in vivo* experiments, we confirmed that ACR suppresses peripheral motor nerve development. It has been reported that hyperglycemia causes disruption of peripheral nerve development in zebrafish (Ennerfelt et al., 2019), but whether ACR-leading effects on peripheral neurogenesis are induced by hyperglycemia is yet to be clarified. Therefore, a hypoglycemic drug PK11195 and ACR scavenger L-carnosine were firstly used for further exploration. Intriguingly, ACR-induced impaired neurogenesis can be reversed by anti-ACR, but not hypoglycemic treatment, suggesting that anti-ACR treatment would be a promising approach to promote neurogenesis, and L-carnosine would be a potential candidate to fulfill the anti-ACR treatment.

Additionally, according to RNA-Seq data, several iron ion metabolic pathways and ferroptosis pathways showed significant upregulation in the ACR treated group. This raised the hypothesis that activated ferroptosis is the possible reason behind impaired peripheral neurogenesis resulting from ACR treatment. To address this question, well-known ferroptosis inducer and ferroptosis inhibitor, Erastin and Ferrostatin, were utilized to determine if normalizing ferroptosis can impede the ACR-leading effects. Interestingly, we observed that ferroptosis inducer, Erastin also led to impaired neurogenesis, which is in line with ACR causing phenotype. Furthermore, ACR and ferroptosis inducer leading alterations can be well-rescued by anti-ferroptosis treatment. Overall, the study affords several important implications. First, it indicates ACR contributes to peripheral neuropathy as a novel factor. Second, it implies that activated ferroptosis resulting from ACR plays an indispensable role in peripheral neuropathy. Last, anti-ACR and anti-ferroptosis would be promising therapeutic approaches for DPN treatment.

In clinics, elevated free-state ACR or ACR-adducts have been determined as prominent characteristics in diabetic patients. It was found that the ACR-lysine adduct (FDP-lysine) was elevated in both type 1 and type 2 diabetic patients’ urine (Daimon et al., 2003; Tsukahara et al., 2003). However, the meaningful role of ACR in the DPN requires further investigations. Based on our study, it would be a prospective strategy to dynamically monitor the endogenous ACR concentration in clinics for filtering people with a pre-diabetic neuropathy state, early diagnosing patients with DPN, and improving long-term prognosis. Lastly, this study also affords an alternative strategy for DPN treatment. General therapy, applying hypoglycemic agents and painkillers benefits a limited number of patients. Whether it would be feasible to treat DPN by using ACR scavenger and ferroptosis inhibitors deserves further study.

Although this study revealed that ACR contributes to peripheral neuropathy by over-activating ferroptosis, some limitations cannot be ignored. First, although it has been confirmed that ACR is able to activate ferroptosis, the precise mechanism behind it remains unknown. Second, the potential pathways and molecular targets involved in ferroptosis and peripheral neurogenesis need further study. Last but not least, clinical studies are necessary to ensure if anti-ACR and/or ferroptosis inhibitors are practical and promising drugs for clinical application.

In conclusion, this study provided evidence for the effects of ACR on peripheral motor nerve development *via* activating ferroptosis in zebrafish, providing a new direction for further research in diabetic neuropathy and therapy.

## Data availability statement

The datasets presented in this study can be found in online repositories. The names of the repository/repositories and accession number(s) can be found below: The RNA-Seq datasets produced in this study are available at GEO (Gene Expression Omnibus, NIH) under the accession address: https://www.ncbi.nlm.nih.gov/geo/query/acc.cgi?acc=GSE168786.

## Ethics statement

The animal study was reviewed and approved by the Local Government and Medical Faculty Mannheim of Heidelberg University.

## Author contributions

HQ designed this study, performed experiments, analyzed data, and wrote the manuscript. KK and CS performed RNA sequencing data analysis. KB, XQ, and SL did incubation experiments. GP performed metabolome studies and analyzed data. JK conceived and designed this study and wrote the manuscript. All authors contributed to the article and approved the submitted version.

## Footnotes

1
www.bgi.com

2
https://biorender.com/

## References

[B1] Azmi S. Petropoulos I. N. Ferdousi M. Ponirakis G. Alam U. Malik R. A. (2019). An update on the diagnosis and treatment of diabetic somatic and autonomic neuropathy. *F1000Res* 8 F1000FacultyRev–186. 10.12688/f1000research.17118.1 30828432 PMC6381801

[B2] Brownlee M. (2001). Biochemistry and molecular cell biology of diabetic complications. *Nature* 414 813–820. 10.1038/414813a 11742414

[B3] Butler B. Acosta G. Shi R. (2017). Exogenous acrolein intensifies sensory hypersensitivity after spinal cord injury in rat. *J Neurol Sci* 379 29–35. 10.1016/j.jns.2017.05.039 28716263

[B4] Callaghan B. C. Gao L. Li Y. Zhou X. Reynolds E. Banerjee M. (2018). Diabetes and obesity are the main metabolic drivers of peripheral neuropathy. *Ann. Clin. Transl. Neurol.* 5 397–405. 10.1002/acn3.531 29687018 PMC5899909

[B5] Callaghan B. C. Little A. A. Feldman E. L. Hughes R. A. (2012). Enhanced glucose control for preventing and treating diabetic neuropathy. *Cochrane Database Syst. Rev.* 6:CD007543. 10.1002/14651858.CD007543.pub2 22696371 PMC4048127

[B6] Callaghan B. C. Price R. S. Feldman E. L. (2015). Distal symmetric polyneuropathy: A review. *JAMA* 314 2172–2181. 10.1001/jama.2015.13611 26599185 PMC5125083

[B7] Callaghan B. C. Xia R. Banerjee M. de Rekeneire N. Harris T. B. Newman A. B. (2016). Metabolic syndrome components are associated with symptomatic polyneuropathy independent of glycemic status. *Diabetes Care* 39 801–807. 10.2337/dc16-0081 26965720 PMC4839175

[B8] Ciura S. Lattante S. Le Ber I. Latouche M. Tostivint H. Brice A. (2013). Loss of function of C9orf72 causes motor deficits in a zebrafish model of amyotrophic lateral sclerosis. *Ann. Neurol.* 74 180–187. 10.1002/ana.23946 23720273

[B9] Cozzi A. Orellana D. I. Santambrogio P. Rubio A. Cancellieri C. Giannelli S. (2019). Stem cell modeling of neuroferritinopathy reveals iron as a determinant of senescence and ferroptosis during neuronal aging. *Stem Cell Rep.* 13 832–846. 10.1016/j.stemcr.2019.09.002 31587993 PMC6893074

[B10] Daimon M. Sugiyama K. Kameda W. Saitoh T. Oizumi T. Hirata A. (2003). Increased urinary levels of pentosidine, pyrraline and acrolein adduct in type 2 diabetes. *Endocr. J.* 50 61–67. 10.1507/endocrj.50.61 12733710

[B11] DeJarnett N. Conklin D. J. Riggs D. W. Myers J. A. O’Toole T. E. Hamzeh I. (2014). Acrolein exposure is associated with increased cardiovascular disease risk. *J. Am. Heart Assoc.* 3:e000934. 10.1161/JAHA.114.000934 25099132 PMC4310380

[B12] Ennerfelt H. Voithofer G. Tibbo M. Miller D. Warfield R. Allen S. (2019). Disruption of peripheral nerve development in a zebrafish model of hyperglycemia. *J. Neurophysiol.* 122 862–871. 10.1152/jn.00318.2019 31268813

[B13] Feldman E. L. Nave K. A. Jensen T. S. Bennett D. L. (2017). New horizons in diabetic neuropathy: Mechanisms, bioenergetics, and pain. *Neuron* 93 1296–1313. 10.1016/j.neuron.2017.02.005 28334605 PMC5400015

[B14] Gong J. Hu S. Huang Z. Hu Y. Wang X. Zhao J. (2020). The requirement of Sox2 for the spinal cord motor neuron development of zebrafish. *Front. Mol. Neurosci.* 13:34. 10.3389/fnmol.2020.00034 32292330 PMC7135881

[B15] Heckler K. Kroll J. (2017). Zebrafish as a model for the study of microvascular complications of diabetes and their mechanisms. *Int. J. Mol. Sci.* 18:2002. 10.3390/ijms18092002 28925940 PMC5618651

[B16] Holmes C. J. Hastings M. (2021). The application of exercise training for diabetic peripheral neuropathy. *J. Clin. Med.* 10:5042. 10.3390/jcm10215042 34768562 PMC8584831

[B17] Jiang C. Jiang L. Li Q. Liu X. Zhang T. Dong L. (2018). Acrolein induces NLRP3 inflammasome-mediated pyroptosis and suppresses migration via ROS-dependent autophagy in vascular endothelial cells. *Toxicology* 410 26–40. 10.1016/j.tox.2018.09.002 30205151

[B18] Kimmel C. B. Ballard W. W. Kimmel S. R. Ullmann B. Schilling T. F. (1995). Stages of embryonic development of the zebrafish. *Dev. Dyn.* 203 253–310. 10.1002/aja.1002030302 8589427

[B19] Lawson N. D. Weinstein B. (2002). In vivo imaging of embryonic vascular development using transgenic zebrafish. *Dev. Biol.* 248 307–318. 10.1006/dbio.2002.0711 12167406

[B20] Lin Y. Chen Z. Tang J. Cao P. Shi R. (2018). Acrolein contributes to the neuropathic pain and neuron damage after ischemic-reperfusion spinal cord injury. *Neuroscience* 384 120–130. 10.1016/j.neuroscience.2018.05.029 29852243

[B21] Lovell M. A. Xie C. Markesbery W. R. (2001). Acrolein is increased in Alzheimer’s disease brain and is toxic to primary hippocampal cultures. *Neurobiol. Aging* 22 187–194. 10.1016/s0197-4580(00)00235-9 11182468

[B22] Lu S. Lyu Z. Wang Z. Kou Y. Liu C. Li S. (2021). Lipin 1 deficiency causes adult-onset myasthenia with motor neuron dysfunction in humans and neuromuscular junction defects in zebrafish. *Theranostics* 11 2788–2805. 10.7150/thno.53330 33456573 PMC7806489

[B23] Murata M. Noda K. Ishida S. (2020). Pathological role of unsaturated aldehyde acrolein in diabetic retinopathy. *Front. Immunol.* 11:589531. 10.3389/fimmu.2020.589531 33193419 PMC7642371

[B24] Nakano T. Windrem M. Zappavigna V. Goldman S. A. (2005). Identification of a conserved 125 base-pair Hb9 enhancer that specifies gene expression to spinal motor neurons. *Dev. Biol.* 283 474–485. 10.1016/j.ydbio.2005.04.017 15913596

[B25] Noiri E. Yamada S. Nakao A. Tsuchiya M. Masaki I. Fujino K. (2002). Serum protein acrolein adducts: Utility in detecting oxidant stress in hemodialysis patients and reversal using a vitamin E-bonded hemodialyzer. *Free Radic. Biol. Med.* 33 1651–1656. 10.1016/s0891-5849(02)01138-3 12488133

[B26] Pagnamenta A. T. Kaiyrzhanov R. Zou Y. Da’as S. I. Maroofian R. Donkervoort S. (2021). An ancestral 10-bp repeat expansion in VWA1 causes recessive hereditary motor neuropathy. *Brain* 144 584–600. 10.1093/brain/awaa420 33559681 PMC8263055

[B27] Pop-Busui R. Boulton A. J. Feldman E. L. Bril V. Freeman R. Malik R. A. (2017). Diabetic neuropathy: A position statement by the american diabetes association. *Diabetes Care* 40 136–154. 10.2337/dc16-2042 27999003 PMC6977405

[B28] Pop-Busui R. Lu J. Brooks M. M. Albert S. Althouse A. D. Escobedo J. (2013). Impact of glycemic control strategies on the progression of diabetic peripheral neuropathy in the bypass angioplasty revascularization investigation 2 diabetes (BARI 2D) Cohort. *Diabetes Care* 36 3208–3215. 10.2337/dc13-0012 23757426 PMC3781573

[B29] Qi H. Schmohl F. Li X. Qian X. Tabler C. T. Bennewitz K. (2021). Reduced acrolein detoxification in akr1a1a zebrafish mutants causes impaired insulin receptor signaling and microvascular alterations. *Adv. Sci. (Weinh)* 8:e2101281. 10.1002/advs.202101281 34278746 PMC8456208

[B30] Sanaye M. Kavishwar M. (2023). Diabetic neuropathy: Review on molecular mechanisms. *Curr. Mol. Med.* 23, 97–110. 10.2174/1566524021666210816093111 34397329

[B31] Song R. Wang R. Shen Z. Chu H. (2022). Sevoflurane diminishes neurogenesis and promotes ferroptosis in embryonic prefrontal cortex via inhibiting nuclear factor-erythroid 2-related factor 2 expression. *Neuroreport* 33 252–258. 10.1097/WNR.0000000000001775 35275882

[B32] Spaas J. Franssen W. M. Keytsman C. Blancquaert L. Vanmierlo T. Bogie J. (2021). Carnosine quenches the reactive carbonyl acrolein in the central nervous system and attenuates autoimmune neuroinflammation. *J. Neuroinflammation* 18:255. 10.1186/s12974-021-02306-9 34740381 PMC8571880

[B33] Stevens J. F. Maier C. (2008). Acrolein: sources, metabolism, and biomolecular interactions relevant to human health and disease. *Mol. Nutr. Food Res.* 52 7–25. 10.1002/mnfr.200700412 18203133 PMC2423340

[B34] Stino A. M. Rumora A. E. Kim B. Feldman E. L. (2020). Evolving concepts on the role of dyslipidemia, bioenergetics, and inflammation in the pathogenesis and treatment of diabetic peripheral neuropathy. *J. Peripher. Nerv. Syst.* 25 76–84. 10.1111/jns.12387 32412144 PMC7375363

[B35] Tsukahara H. Sekine K. Uchiyama M. Kawakami H. Hata I. Todoroki Y. (2003). Formation of advanced glycosylation end products and oxidative stress in young patients with type 1 diabetes. *Pediatr. Res.* 54 419–424. 10.1203/01.PDR.0000076662.72100.74 12761359

[B36] Uchida K. (2000). Role of reactive aldehyde in cardiovascular diseases. *Free Radic Biol. Med.* 28 1685–1696. 10.1016/s0891-5849(00)00226-4 10946210

[B37] Vaccaro A. Patten S. A. Ciura S. Maios C. Therrien M. Drapeau P. (2012). Methylene blue protects against TDP-43 and FUS neuronal toxicity in C. elegans and D. rerio. *PLoS One* 7:e42117. 10.1371/journal.pone.0042117 22848727 PMC3407135

[B38] Wiggenhauser L. M. Kroll J. (2019). Vascular damage in obesity and diabetes: Highlighting links between endothelial dysfunction and metabolic disease in zebrafish and man. *Curr. Vasc. Pharmacol.* 17 476–490. 10.2174/1570161116666181031101413 30378499

[B39] Wiggenhauser L. M. Qi H. Stoll S. J. Metzger L. Bennewitz K. Poschet G. (2020). Activation of retinal angiogenesis in hyperglycemic pdx1 (-/-) zebrafish mutants. *Diabetes* 69 1020–1031. 10.2337/db19-0873 32139597

[B40] Zakin E. Abrams R. Simpson D. M. (2019). Diabetic neuropathy. *Semin. Neurol.* 39 560–569. 10.1055/s-0039-1688978 31639839

[B41] Zhang X. Lai Y. McCance D. R. Uchida K. McDonald D. M. Gardiner T. A. (2008). Evaluation of N (epsilon)-(3-formyl-3,4-dehydropiperidino)lysine as a novel biomarker for the severity of diabetic retinopathy. *Diabetologia* 51 1723–1730. 10.1007/s00125-008-1071-3 18587559

[B42] Zhao J. Posa D. K. Kumar V. Hoetker D. Kumar A. Ganesan S. (2019). Carnosine protects cardiac myocytes against lipid peroxidation products. *Amino. Acids* 51 123–138. 10.1007/s00726-018-2676-6 30449006 PMC6377314

