# Supplementary figures and images for: Acrolein-inducing ferroptosis contributes to impaired peripheral neurogenesis in zebrafish
Source: Front Neurosci. 2023 Jan 12;16:1044213. doi: 10.3389/fnins.2022.1044213 (PMC9877442; doi:10.3389/fnins.2022.1044213)

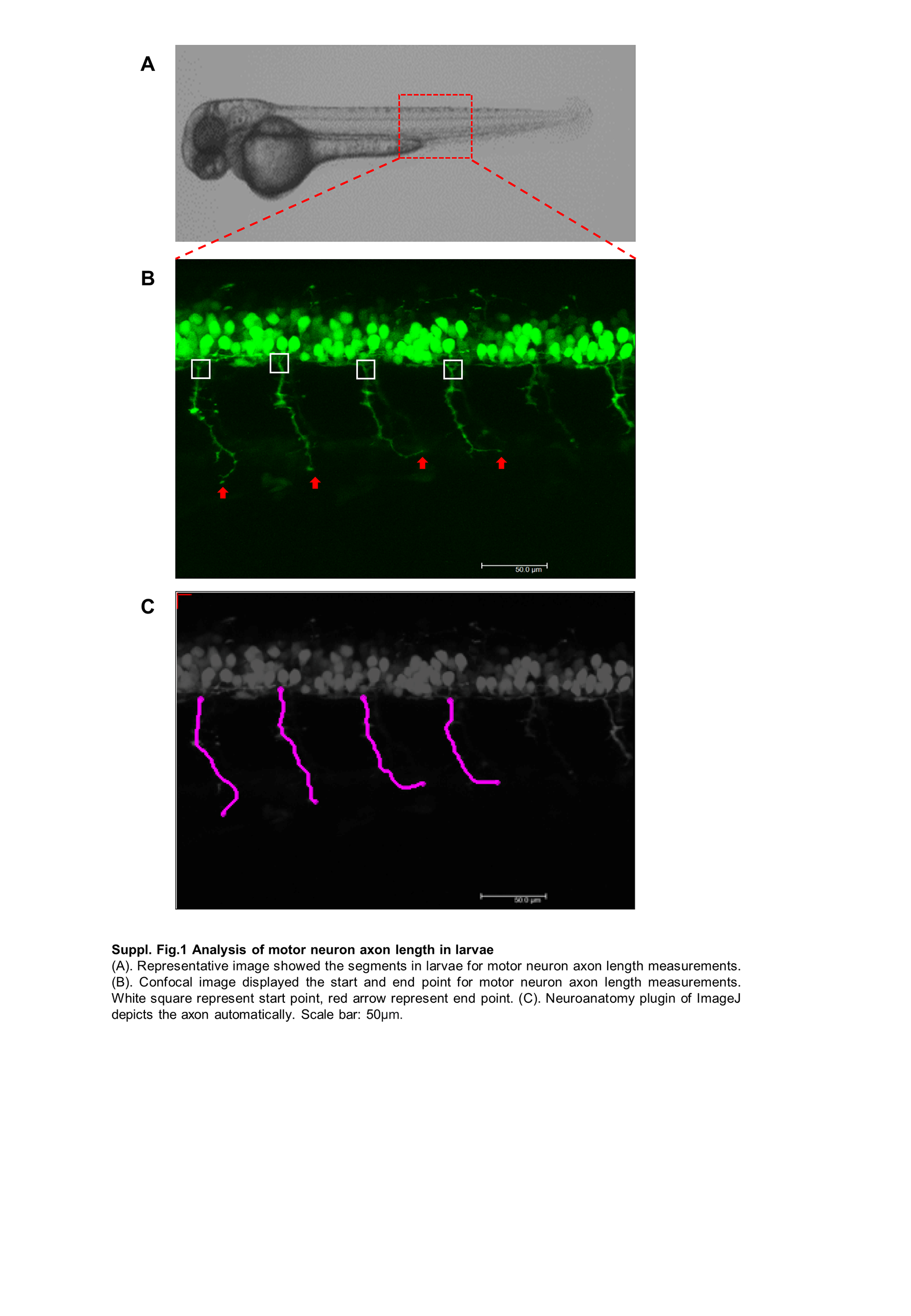

Supplement: Supplementary file 1 [file Image_1.TIF]

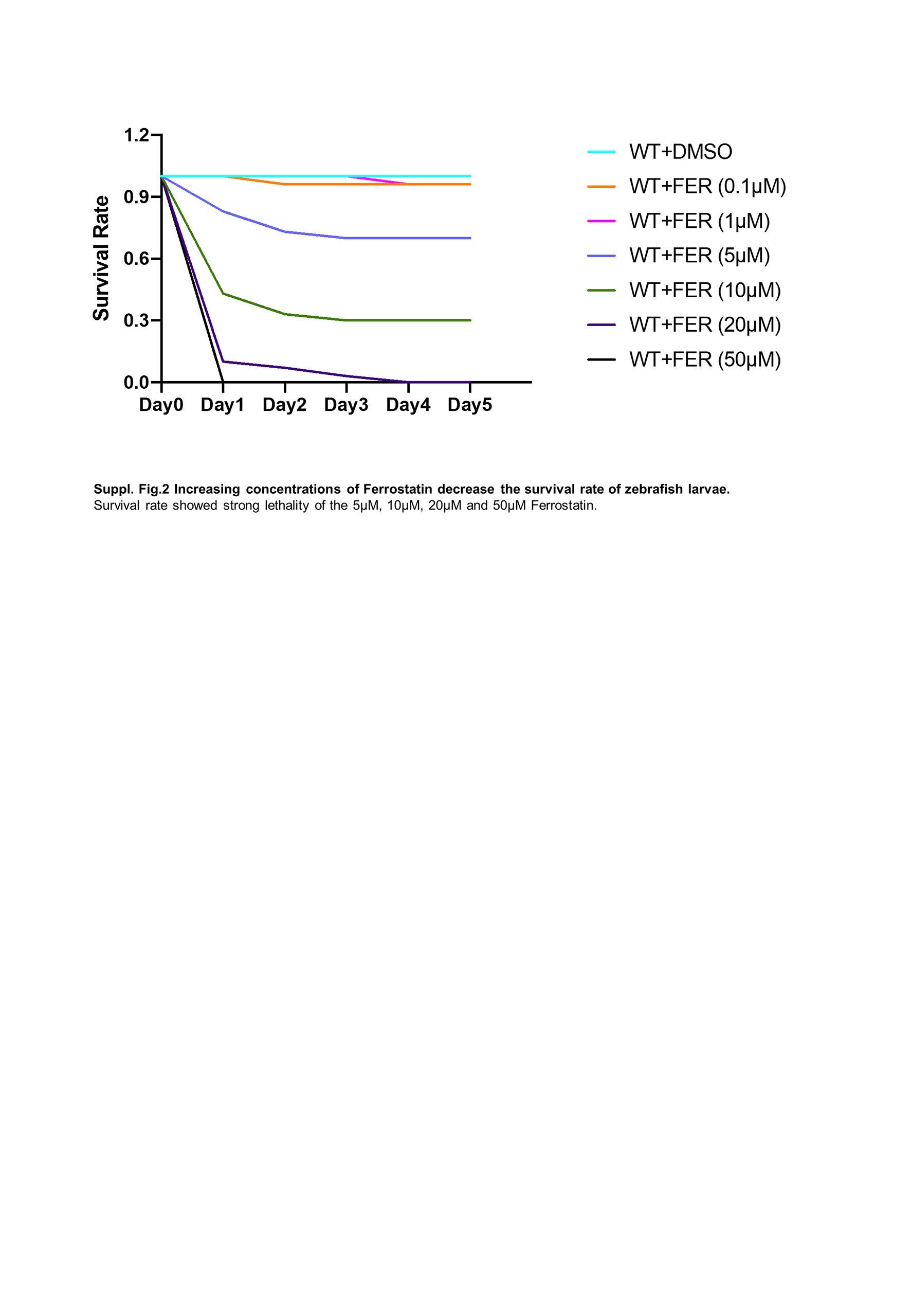

Supplement: Supplementary file 2 [file Image_2.TIF]

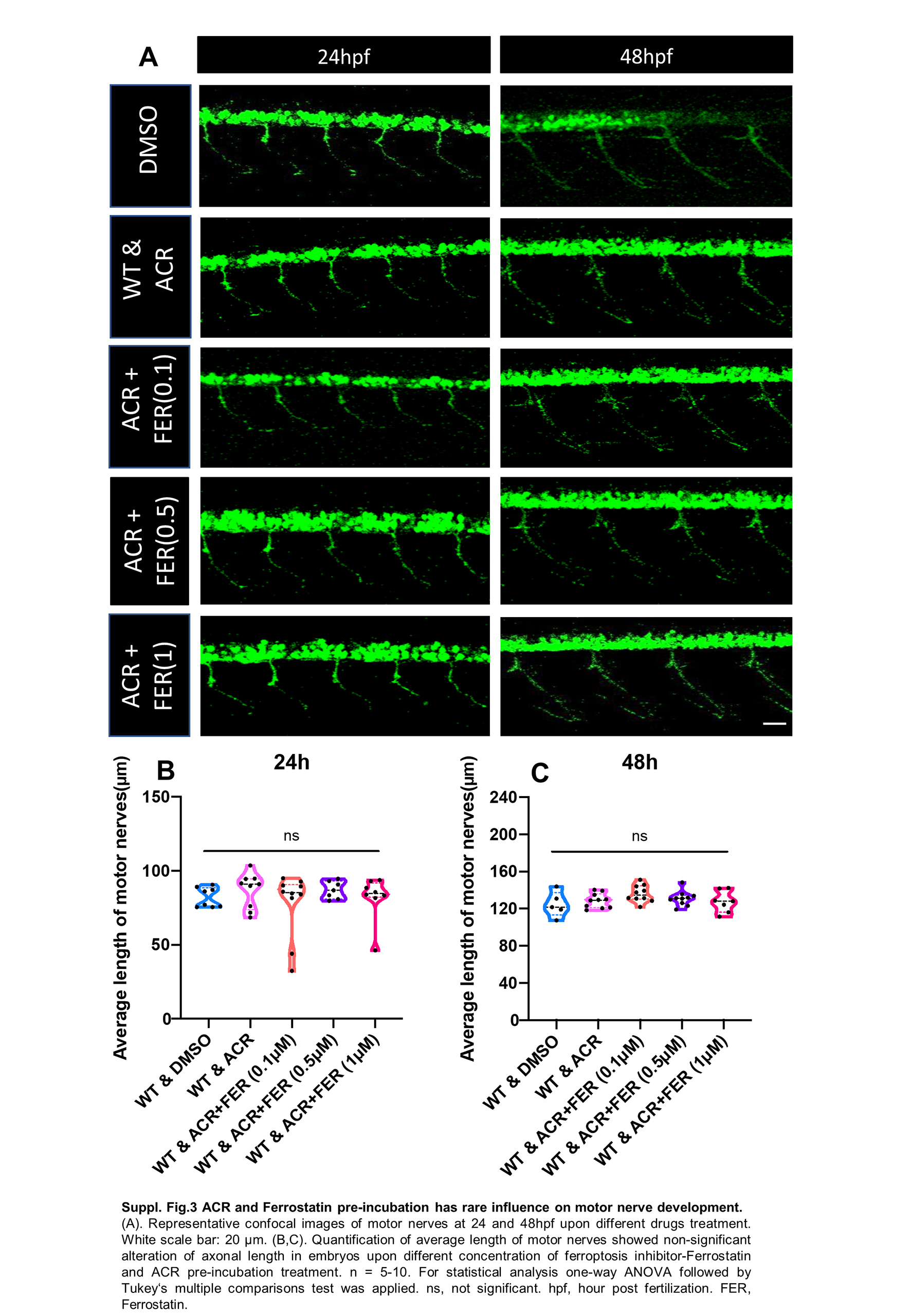

Supplement: Supplementary file 3 [file Image_3.TIF]
